# Asthma symptoms, spirometry and air pollution exposure in schoolchildren in an informal settlement and an affluent area of Nairobi, Kenya

**DOI:** 10.1136/thorax-2023-220057

**Published:** 2023-06-06

**Authors:** Hellen Meme, Evans Amukoye, Cressida Bowyer, Jeremiah Chakaya, Darpan Das, Ruaraidh Dobson, Ulrike Dragosits, Jonathan Fuld, Cindy Gray, Matthew Hahn, Richard Kiplimo, Maia Lesosky, Miranda M Loh, Jean McKendree, Kevin Mortimer, Amos Ndombi, Louis Netter, Angela Obasi, Fred Orina, Clare Pearson, Heather Price, Jennifer K Quint, Sean Semple, Marsailidh Twigg, Charlotte Waelde, Anna Walnycki, Melaneia Warwick, Jana Wendler, Sarah E West, Michael Wilson, Lindsay Zurba, Graham Devereux

**Affiliations:** 1 Centre for Respiratory Disease Research, Kenya Medical Research Institute, Nairobi, Kenya; 2 Faculty of Creative and Cultural Industries, University of Portsmouth, Portsmouth, UK; 3 Institute of Occupational Medicine, Edinburgh, UK; 4 Institute for Social Marketing and Health, University of Stirling, Stirling, UK; 5 UK Centre for Ecology & Hydrology, Penicuik, UK; 6 Respiratory Medicine, Cambridge University Hospitals NHS Foundation Trust, Cambridge, UK; 7 School of Social and Political Sciences, University of Glasgow, Glasgow, Glasgow, UK; 8 Theatre for Development Facilitator, Folkstone, UK; 9 Global Health Trials Unit, Liverpool School of Tropical Medicine, Liverpool, UK; 10 Department of Environment and Geography, University of York, York, UK; 11 Department of Medicine, University of Cambridge, Cambridge, UK; 12 Department of Medicine, College of Health Sciences University of KwaZulu-Natal, Durban, South Africa; 13 Department of International Public Health, Liverpool School of Tropical Medicine, Liverpool, UK; 14 Axess Sexual Health, Liverpool University Hospitals NHS Foundation Trust, Liverpool, Liverpool, UK; 15 Biological and Environmental Sciences, University of Stirling, Stirling, UK; 16 NHLI, Imperial College London, London, UK; 17 Centre for Dance Research, Coventry University, Coventry, UK; 18 International Institute for Environment and Development, London, UK; 19 School of Design & Creative Arts, Loughborough University, Loughborough, UK; 20 Playfuel Games Ltd, Manchester, UK; 21 Education for Health Africa, Durban, South Africa; 22 Department of Clinical Sciences, Liverpool School of Tropical Medicine, Liverpool, UK

**Keywords:** asthma, asthma epidemiology, paediatric asthma

## Abstract

**Background:**

Although 1 billion people live in informal (slum) settlements, the consequences for respiratory health of living in these settlements remain largely unknown. This study investigated whether children living in an informal settlement in Nairobi, Kenya are at increased risk of asthma symptoms.

**Methods:**

Children attending schools in Mukuru (an informal settlement in Nairobi) and a more affluent area (Buruburu) were compared. Questionnaires quantified respiratory symptoms and environmental exposures; spirometry was performed; personal exposure to particulate matter (PM_2.5_) was estimated.

**Results:**

2373 children participated, 1277 in Mukuru (median age, IQR 11, 9–13 years, 53% girls), and 1096 in Buruburu (10, 8–12 years, 52% girls). Mukuru schoolchildren were from less affluent homes, had greater exposure to pollution sources and PM_2.5_. When compared with Buruburu schoolchildren, Mukuru schoolchildren had a greater prevalence of symptoms, ‘current wheeze’ (9.5% vs 6.4%, p=0.007) and ‘trouble breathing’ (16.3% vs 12.6%, p=0.01), and these symptoms were more severe and problematic. Diagnosed asthma was more common in Buruburu (2.8% vs 1.2%, p=0.004). Spirometry did not differ between Mukuru and Buruburu. Regardless of community, significant adverse associations were observed with self-reported exposure to ‘vapours, dusts, gases, fumes’, mosquito coil burning, adult smoker(s) in the home, refuse burning near homes and residential proximity to roads.

**Conclusion:**

Children living in informal settlements are more likely to develop wheezing symptoms consistent with asthma that are more severe but less likely to be diagnosed as asthma. Self-reported but not objectively measured air pollution exposure was associated with increased risk of asthma symptoms.

**WHAT IS ALREADY KNOWN ON THIS TOPIC**

- An estimated 350–500 million children live in informal settlements (also known as slums).
- Although it is believed that children in informal settlements are at increased risk of asthma, this appears to be based on the associations between childhood asthma and poverty and/or air pollution and not comparative studies of children living within and outside informal settlements.

**WHAT THIS STUDY ADDS**

- This study appears to be the first to compare asthma in children living in an informal settlement with children living in a more affluent area of the same city.
- When compared with other urban children, children living in informal settlements have a clinically important increased risk of symptoms consistent with asthma that are also more severe, and less likely to be diagnosed as asthma.
- Indoor and outdoor sources of air pollution are associated with this increased risk.

**HOW THIS STUDY MIGHT AFFECT RESEARCH, PRACTICE OR POLICY**

- There is a need to identify, diagnose and treat asthma symptoms in children living in informal settlements.
- In the medium term to long term, sources of indoor and outdoor air pollution need to be addressed.

## Introduction

A feature of current rapid global urbanisation is the development of informal settlements (also known as slums).^1^ In 2005, about 15% of the world’s population (1 billion people) lived in informal settlements and this is projected to increase to 32% (2 billion people) by 2030.^2 3^ The majority of informal settlements are in low-income to middle-income countries (LMICs) with sub-Saharan Africa (SSA) having the largest proportion of urban residents living in informal settlements (72%).^3^ The proliferation of informal settlements in SSA has been attributed to rapid urbanisation occurring at a time of poor economic performance and inadequate urban governance.

Informal settlements are characterised by poor environmental conditions, hazardous locations, overcrowding, substandard housing, unemployment and poverty and the consequences of these for the health of residents in informal settlements has led to calls for specific research into this issue.^1 2^ An estimated 350–500 million children live in informal settlements and despite improving childhood mortality in recent years, 0–5 years mortality in informal settlements remains higher than rural and urban areas, with pneumonia and diarrhoea being the leading causes of childhood morbidity and mortality.^4–7^ Asthma is the most common non-communicable disease of childhood being responsible for a considerable morbidity and mortality burden in LMICs.^8 9^ While it has been suggested that living in informal settlements is associated with an increased risk of asthma,^1^ this is primarily based on considerations of the associations between poverty and asthma, and air pollution and asthma,^10 11^ rather than studies comparing children living in informal settlements with other urban children.

We have carried out a cross-sectional observational study in Nairobi, Kenya, comparing the respiratory characteristics and environmental exposures of children attending schools within the informal settlement of Mukuru with children attending schools in the nearby more affluent residential area of Buruburu. The aims of the Tupumue (Swahili for ‘let us breathe’) study were to investigate whether there is a difference in asthma symptoms, other respiratory symptoms and lung function between the schoolchildren of Mukuru and Buruburu and to explore whether indoor/outdoor air pollution is associated with asthma symptoms, other respiratory symptoms and lung function. The focus on air pollution was requested by both communities who are greatly worried by the possible impact of air pollution on their children’s health.

## Materials and methods

### Study setting

Kenya is a lower middle-income country with a population of 55 million and per capita income of US$1816/year. The capital city, Nairobi, is typical of the rapid urbanisation in SSA with 60%–70% of residents living in over 100 informal settlements.^6^ Mukuru is one of the largest informal settlements in Kenya occupying 450 acres in Nairobi’s industrial area.^12^ Population estimates range from 300 to 700 000. Mukuru is overcrowded and polluted by unregulated emissions from local industry, traffic fumes, dust and the burning of household and commercial waste. There is widespread poverty, poor sanitation and a lack of basic amenities. Mukuru has many health issues, but community engagement and clinical studies highlight a high burden of respiratory disease.^13^ The nearby suburb of Buruburu is a large planned residential area established in the 1970s and 1980s as an owner-occupier housing project consisting of 5000 three-bedroom single dwelling units. Buruburu remains largely owner-occupied, although some parts have been extended for rental purposes, and is mainly inhabited by business people and professionals. Community engagement highlights major concerns about traffic emissions.

### Community involvement and sensitisation

To maximise study relevance and acceptance in the two communities, co-creation workshops were held in each community to inform all aspects of study design, data collection and communication. These workshops were attended by children, parents, teachers, community workers, chiefs and County/National Government representatives. At these workshops, attendees recommended a community sensitisation programme in the form of a series of public-based and school-based activities to raise awareness of the project, its focus and to allay concerns among community residents.^14^ Academics, arts practitioners, residents and community artists co-created a suite of innovative, inclusive and culturally relevant sensitisation tools to explain the project rationale and methods to the adults and children of both communities. These included a theatre show dramatising the process of data collection, dance, games, a song, puppetry, visual arts and a music video (https://www.youtube.com/@tupumue5458). The sensitisation programme was delivered by local Tupumue community champions to audiences in schools, churches and community venues.

### Recruitment

All children aged ≤18 years attending schools in Mukuru or Buruburu were eligible. Participating schools in each community were randomly selected from a sampling frame of all public and private sector schools in that area, stratified by primary/secondary status, using a computer-based sampling algorithm. For each chosen school, a class from each year group was randomly selected from a sampling frame of all classes in year groups using a procedure involving the throw of a die witnessed by the head teacher. The parents/guardians of the children in identified classes were approached to inform them of the study and to obtain consent for their children to participate.

### Questionnaires

Field workers administered questionnaires to parents/guardians of children aged ≤12 years and to the children aged ≥13 years. The field workers read out the questions in the respondents’ preferred language (Kiswahili or English), with responses entered directly into electronic tablets.

The questionnaires included:

1. Demographics, school, age, sex, household asset-based wealth score.^15^
2. Respiratory symptoms, for example, wheezing, ‘trouble breathing’, asthma, asthma management and health service use.^16 17^
3. Self-report of environmental exposures, for example, exposure to traffic and domestic pollution sources, burning of waste and household energy use.^17^

The questions asked are included in the online supplemental material.

10.1136/thorax-2023-220057.supp1 Supplementary data

### Spirometry

Children performed spirometry using the EasyOn Spirometer (NDD Medizintechnik AG, Switzerland) with on-screen incentive software. Nine technicians trained by Education for Health Africa and certified by the Pan African Thoracic Society (PATS) conducted spirometry in accordance with American Thoracic Society/European Respiratory Society recommendations.^18^ Up to eight forced exhalation manoeuvres were performed while sitting and wearing nose clips. An internal and external assessor reviewed all blows, with measurements graded A–C for acceptability and repeatability being selected for analysis. Children with forced expiratory volume in 1 s (FEV_1_) ≥70% predicted were asked to undertake a 6 min run test (running for 6 min at jogging pace).^19^ The highest acceptable/repeatable FEV_1_ at 0, 5 and 10 minu after exercise was recorded.

### Air quality monitoring

To estimate children’s personal exposure to fine particulate matter (PM_2.5_), a subset of about 100 children from each community participated in detailed air quality monitoring (AQM) of their homes. AQM was also undertaken within participating schools and outdoor settings (eg, local meeting places, roadsides) identified by children as places where they spent their leisure time. PM_2.5_ concentrations were measured using PurpleAir PA-II-SD sensors (PurpleAir, Draper, Utah, USA).

Twenty-four-hour time-weighted average (TWA) personal PM_2.5_ exposure was calculated using:

$$PM_{TWA}=(T_{Roadside}+T_{Travel})\times PM_{Outdoor}+T_{School}\times PM_{School}+T_{Home}\times PM_{Home}$$

Time spent at home (*T_Home_*) was calculated using reported values. Time spent at school was assigned by child’s age and appropriate school times. Time at roadside and spent travelling was reported in the questionnaire.

Associations between the PM_2.5_ 24-hour TWA concentrations for the AQM subsample and a range of possible exposure determinants identified from the environmental exposure questionnaire were examined using linear regression. The final regression model related measured TWA personal PM_2.5_ exposure in the subset of children to the statistically significant variables: child age, school location (Mukuru/Buruburu), adult smoker in the home and the primary home cooking fuel. This model was then applied to the data from all participating children to estimate the 24-hour TWA PM_2.5_ exposure for each child in the entire dataset.

### Sample size

In each community, we aimed to collect symptom and spirometry data for 1000 and 800 children, respectively. The primary determinant of sample size was pragmatic, being the maximum considered possible given the available resources because there were no contemporary local respiratory symptom or spirometry data available for children aged ≤18 years. With symptom data for 1000 children in each community, the study had 80% power, with α=0.05, to detect a difference of ±5% for those symptoms with prevalence of 20% (15.2%–25.2%) and ±3% (2.5%–8.2%) for those symptoms with prevalence of 5%. With spirometry data for 800 children in each community, the study had 80% power, with α=0.05, to detect z-score differences of ±0.14.

### Statistics

As in many epidemiological studies of asthma prevalence in children, asthma prevalence estimates were based on the responses to the questions ‘Has child had wheezing or whistling in the chest in the past 12 months?’ (current wheeze), and ‘Has child ever had asthma?’ (ever asthma), with asthma severity being based on responses to questions enquiring about number of wheezing attacks, speech limiting wheeze and sleep disturbance by wheeze (all in the last 12 months).^20 21^ Other respiratory symptoms of interest included ‘trouble breathing’ (Does child ever have trouble with his/her breathing?), ‘cough’ (dry cough at night, in absence of cold/chest), ‘days off school with breathing problems’ and ‘seeking urgent medical attention for breathing problems’. The spirometry parameters were FEV_1_, forced vital capacity (FVC) and the ratio (FEV_1_/FVC). FEV_1_ and FVC were expressed as z-scores using Global Lung Initiative 2012 reference equations for African-American ethnicity in the absence of more appropriate Kenyan reference equations.^22^ Percentage exercise-induced bronchoconstriction (EIB) was calculated in the usual way using the lowest FEV_1_ recorded at 0, 5 or 10 min postexercise.^19^ The air pollution exposures of interest were estimated 24-hour TWA PM_2.5_, exposure to vapours, dusts, gases or fumes >15 hours/week, burning of refuse within sight of the home, residential proximity to a major road, tobacco smoking within the home, burning of mosquito coils and cooking fuels.

Characteristics are summarised by community and overall. Unadjusted and adjusted analyses were conducted to estimate exposure outcome associations. Adjusted analysis included adjusting exposure measurements for age, sex, household asset wealth score and community. Hosmer-Lemeshow tests were used to confirm goodness-of-fit of logistic models. Analysis used the statistical package R, p<0.05 was considered statistically significant.^23^

## Results

Recruitment took place between January 2020 and November 2021, with a 14-month suspension between March 2020 and April 2021 because of COVID-19. In total, 3450 children aged 4–18 years were invited to participate, of whom 2373 (69%) took part. In Mukuru, 1277 children (64%) of 1980 children attending 5 primary and 1 secondary school(s) were recruited; and in Buruburu, 1096 children (75%) were recruited from 1470 children attending 4 primary and 2 secondary schools. When compared with Buruburu schoolchildren, Mukuru schoolchildren were older, came from less affluent homes and were more likely to report being exposed to ‘vapours, dusts, gases, fumes’, adult smokers in the home, burning mosquito coils and refuse burning within sight of the home (table 1). The predominant reported cooking fuel in Buruburu was liquid petroleum gas, whereas kerosene/paraffin and solid fuels/open fires were reported more frequently in Mukuru. More homes in Buruburu were close to major roads than in Mukuru. Estimated PM_2.5_ exposures were higher in Mukuru (median 39 µg/m³, IQR 35–43) than in Buruburu (median 22 µg/m³, 20–25). Although numbers were small (<10), there were no significant differences in the number of children who smoked or who had ever been diagnosed with tuberculosis or HIV in each community.

**Table 1:** Socio-economic, environmental and communicable disease characteristics of participating children attending schools in Mukuru and Buruburu

	Buruburu (n=1096)	Mukuru (n=1277)	P value
Girls (n, %)	567 (51.7%)	673 (52.7%)	0.65
Age (median, IQR)	10 (8–12)	11 (9–13)	<0.001
Household assets owned (median, IQR)^15^	6 (3–7)	3 (2–3)	<0.001
Estimated 24-hour time-weighted average PM_2.5_ (µg/m^3^) mean (95% CI)	22.3 (22.1 to 22.5)	39.9 (39.5 to 40.2)	<0.001
Exposed vapours, dusts, gases, fumes >15 hours/week (n, %)	565 (54.6%)	815 (70.3%)	<0.001
Refuse burnt within sight of home (n, %)	330 (30.2%)	485 (38.1%)	<0.001
Proximity of home to major road (n, %)			
<100 m	569 (51.9%)	554 (43.4%)	<0.001
100–500 m	397 (36.2%)	398 (31.2%)	
>500 m	130 (11.9%)	325 (25.5%)	
Smoker in the home (n, %)	90 (8.2%)	159 (12.5%)	<0.001
Burn mosquito coils in home (n, %)	197 (18.0%)	342 (26.8%)	<0.001
Primary cooking fuel in home (n, %)			
Electric/Solar	8 (0.7%)	17 (1.3%)	<0.001
Gas	850 (77.6%)	598 (46.8%)	
Kerosene/Paraffin	201 (18.3%)	544 (42.6%)	
Solid fuel/open fire	37 (3.4%)	118 (9.2%)	

The questions asked are documented in online supplemental file.

PM, particulate matter.

### Respiratory symptoms

When compared with Buruburu schoolchildren, Mukuru schoolchildren were more likely to experience the symptoms of ‘current wheeze’ and ‘trouble breathing’, and these appeared to be more severe, with more speech and sleep limiting wheeze, more days off school and more acute doctor consultations for breathing problems (table 2). However, Mukuru schoolchildren were less likely to be diagnosed with asthma and to be using inhaled therapies than Buruburu schoolchildren. Dry nocturnal cough was similar in Mukuru and Buruburu schoolchildren.

**Table 2:** Respiratory symptoms and diagnoses of participating schoolchildren children in Mukuru and Buruburu

Symptom*	Buruburu (n=1096)	Mukuru (n=1277)	P value
Wheeze in last 12 months (n, %)	70 (6.4%)	120 (9.5%)	0.007
Number of wheezing attacks in past 12 months (n, %)			
None	1023 (94.0%)	1155 (91.2%)	0.02†
1–3	46 (4.2%)	84 (6.6%)	
≥4	19 (1.7%)	28 (2.2%)	
Sleep disturbed by wheeze in past 12 months (n, %)	46 (4.2%)	88 (6.9%)	0.005
Wheezing severe enough to limit speech in past 12 months (n, %)	24 (2.2%)	46 (3.6%)	0.04
Child ever had asthma (n, %)	33 (3.0%)	16 (1.3%)	0.003
Doctor confirmed asthma (n, %)	31 (2.8%)	15 (1.2%)	0.004
Used inhalers for breathing problems in past 12 months (n, %)	17 (1.6%)	≤5 (≤0.4%)	0.001
Dry cough at night in past 12 months (n, %)	135 (12.4%)	152 (12.0%)	0.79
Trouble with breathing (n, %)	138 (12.6%)	208 (16.3%)	0.01
Days off school because of breathing problems in past 12 months (n, %)			
None	1042 (95.1%)	1182 (92.6%)	0.01†
1–3	35 (3.2%)	57 (4.5%)	
≥4	19 (1.7%)	38 (3.0%)	
Times taken urgently to doctor because of breathing problems, in past 12 months (n, %)			
None	1026 (93.6%)	1166 (91.3%)	0.02†
1–3	64 (5.8%)	94 (7.4%)	
≥4	6 (0.5%)	17 (1.3%)	
Child ever diagnosed with pneumonia (n, %)	181 (16.7%)	308 (24.3%)	<0.001
Chest infection in first year of life (n, %)	113 (10.3%)	166 (13.0%)	0.04

*The questions asked are documented in online supplemental file.

†P linear trends.

After adjustment for age, sex, household asset wealth score and community, ‘current wheeze’ was adversely associated with exposure to ‘vapours, dusts, gases, fumes’, refuse burning within sight of the home, adult smokers in the home and burning of mosquito coils in the home (table 3). After similar adjustment, ‘trouble breathing’ was adversely associated with exposure to ‘vapours, dusts, gases, fumes’, living <500 m from a major road and adult smokers in the home.

**Table 3:** Associations between the symptoms of wheeze and trouble breathing and sociodemographic and environmental exposures

	Wheeze in last 12 months (n=190)				Trouble breathing (n=346)			
	Unadjusted OR (95% CI)	P value	Adjusted OR* (95% CI)*	P value	Unadjusted OR (95% CI)	P value	Adjusted OR* (95% CI)	P value
Estimated PM_2.5_ exposure (/10µg/m^3^)	1.27 (1.10 to 1.46)	0.001	0.96 (0.66 to 1.39)	0.82	1.21 (1.09 to 1.35)	0.001	1.08 (0.81 to 1.43)	0.61
Exposed vapours, dusts, gases, fumes >15 hours/week	2.11 (1.48 to 3.01)	<0.001	2.00 (1.40 to 2.87)	<0.001	1.58 (1.23 to 2.04)	0.001	1.51 (1.17 to 1.97)	0.002
Refuse burnt within sight of home	1.50 (1.10 to 2.03)	0.01	1.47 (1.09 to 2.00)	0.01	1.26 (0.99 to 1.59)	0.06	1.23 (0.97 to 1.56)	0.08
Proximity of home to major road								
<100 m	1.11 (0.74 to 1.66)	0.61	1.19 (0.79 to 1.80)	0.41	1.79 (1.26 to 2.52)	0.001	1.92 (1.35 to 2.74)	<0.001
100–500 m	0.99 (0.64 to 1.53)	0.96	1.07 (0.69 to 1.66)	0.76	1.57 (1.09 to 2.27)	0.02	1.70 (1.17 to 2.46)	0.005
>500 m	1		1		1		1	
Smoker in the home	1.71 (1.13 to 2.58)	0.003	1.33 (1.09 to 2.11)	0.02	1.64 (1.18 to 2.28)	0.003	1.58 (1.13 to 2.02)	0.007
Burn mosquito coils in home	1.56 (1.12 to 2.16)	0.01	1.51 (1.02 to 2.03)	0.04	1.28 (0.99 to 1.67)	0.06	1.25 (0.96 to 1.63)	0.10
Main cooking fuel								
Electric/Solar	1		1		1		1	
Gas	1.68 (0.23 to 12.5)	0.61	1.60 (0.21 to 12.1)	0.65	1.59 (0.37 to 6.81)	0.53	1.53 (0.35 to 6.56)	0.57
Kerosene/Paraffin	3.08 (0.41 to 23.1)	0.27	2.97 (0.40 to 22.4)	0.29	2.57 (0.60 to 11.0)	0.20	2.60 (0.60 to 11.2)	0.20
Solid fuel/open fire	1.85 (0.23 to 15.0)	0.57	1.79 (0.22 to 14.5)	0.59	2.99 (0.67 to 13.4)	0.15	2.98 (0.67 to 13.3)	0.15

*Adjusted for children’s age (years), sex (boy vs girl), household asset wealth score (0–10) and community (Mukuru vs Buruburu).

PM, particulate matter.

For Mukuru schoolchildren, 1113 (87%) lived in Mukuru, 164 lived elsewhere (not Buruburu). For Buruburu schoolchildren, 12 (1.1%) lived in Mukuru, 163 (14.9%) lived in Buruburu and 921 (84.0%) lived elsewhere (not Mukuru). Sensitivity analyses reallocating Mukuru children attending schools in Buruburu to Mukuru had minimal effect, ‘current wheeze’ declined from 6.4% to 6.2% in Buruburu and increased from 9.5% to 9.6% in Mukuru and changes of similar magnitude were seen for ‘trouble breathing’.

### Spirometry

In total, 1655 children attempted spirometry, of which 1622 were acceptable/reproducible, 718 children did not attempt spirometry, mainly because the children could not be identified for spirometry testing post-COVID-19. Of the children with acceptable/reproducible spirometry, 1575 did the 6 min run test. Although children unable/unwilling to provide acceptable spirometry were younger, they were very similar demographically and symptomatically to those providing acceptable spirometry (table 4). Although FEV_1_ and FVC were not associated with wheeze or asthma, FEV_1_/FVC z-score was lower (difference −0.39, 95% CI 0.04 to 0.74, p=0.03) and EIB was higher (difference 4.20%, 95% CI 1.05 to 7.35, p=0.01) in children with ‘ever asthma’. There were no significant differences in FEV_1_, FVC, FEV_1_/FVC or EIB between Mukuru and Buruburu schoolchildren (table 5). Analyses relating spirometry parameters to air pollution exposures identified no significant associations after adjustment for age, sex, household wealth asset score and community.

**Table 4:** Characteristics of children with and without acceptable/reproducible spirometry

	Acceptable spirometry (n=1622)	No acceptable spirometry (n=751)	P value
Schooled in Mukuru	872 (53.8%)	405 (53.9%)	0.94
Age (median, IQR)	11 (8,13)	10 (7, 13)	<0.001
Sex (female n, %)	845 (52.1%)	395 (52.6%)	0.82
Wealth/Asset score (median, IQR)	3 (3, 5)	3 (3, 6)	0.05
Wheeze in last 12 months (n, %)	127 (7.9%)	63 (8.4%)	0.66
Doctor diagnosed asthma (n, %)	26 (1.6%)	20 (2.7%)	0.11
Trouble breathing (n, %)	238 (14.7%)	108 (14.4%)	0.85

**Table 5:** Spirometric indices in Mukuru and Buruburu schoolchildren

	Buruburu (n=750)	Mukuru (n=872)	P value
FEV_1_ z-score mean (95% CI)	0.289 (0.214 to 0.363)	0.341 (0.270 to 0.412)	0.31
FEV_1_ z-score <LLN	20 (2.7%)	23 (2.6%)	0.54
FVC z-score mean (95% CI)	0.238 (0.167 to 0.308)	0.322 (0.252 to 0.391)	0.10
FVC z-score <LLN	19 (2.5%)	20 (2.3%)	0.87
FEV_1_/FVC z-score mean (95% CI)	0.075 (0.004 to 0.146)	0.027 (−0.034 to 0.089)	0.32
FEV_1_/FVC z-score <LLN	28 (3.7%)	23 (2.6%)	0.25
EIB (%) mean (95% CI)	4.03 (3.27 to 4.79) (n=727)	3.72 (3.30 to 4.14) (n=848)	0.47

EIB, exercise-induced bronchoconstriction; FEV_1_, forced expiratory volume in 1 s; FVC, forced vital capacity; LLN, lower limit of normal.

## Discussion

We believe this to be the first study comparing asthma symptoms and exposure to air pollution in children living in an informal settlement with those from more affluent residential areas in an LMIC. We found that children attending schools in an informal settlement have higher prevalence of ‘current wheeze’ than children attending schools in a more affluent nearby residential area, and that wheezing symptoms were more severe. Moreover, the children attending schools in the informal settlement were less likely to be diagnosed with asthma and to be using inhaled asthma medications. In regression analyses, significant adverse associations were observed between ‘current wheeze’ and self-reported exposure to ‘vapours, dusts, gases, fumes’, use of mosquito coils, adult smoker(s) in the home and refuse burning within sight of the home.

Although a minority of children (14.9%) attending schools in the more affluent Buruburu suburb lived locally in Buruburu, most lived in adjacent residential areas and were from more affluent homes than the children attending schools in Mukuru, 87% of whom lived locally. Objective measurements of PM_2.5_ concentrations from homes and schools in the two areas found that Mukuru schoolchildren had, on average, close to double the personal PM_2.5_ exposure of Buruburu schoolchildren: PM_2.5_ exposures in both communities greatly exceeded the WHO guidance limit of 15 μg/m^3^ (24 hour average).^24^ Mukuru schoolchildren were more likely than Buruburu schoolchildren to report being exposed to ‘vapours, dusts, gases, fumes’, nearby refuse burning and in the home, adult smokers, burning mosquito coils and solid cooking fuels, while Buruburu schoolchildren were more likely to live close to major roads (an exposure associated with an increased risk of asthma).

Previous studies of the respiratory health of children living in informal settlements have reported that communicable diseases, such as pneumonia and acute respiratory tract infections, are leading causes of childhood morbidity and mortality.^6 7^ Although it is widely acknowledged that asthma is the major non-communicable disease of childhood with considerable health and societal burden in LMICs,^8 9^ we are not aware of any studies that have compared the prevalence of asthma symptoms in children living in informal settlements with children who do not. WHO has highlighted the absence of air pollution data within LMIC cities with sufficient granularity to compare air pollution exposures and consequences for health for the residents of formal and informal settlement areas of cities.^2^ In our study, reported episodes of pneumonia, early life respiratory infections and air pollution levels were higher in the informal settlement than in adjacent more affluent residential areas. In addition, we found that, when compared with more affluent Buruburu schoolchildren, Mukuru schoolchildren experience more ‘current wheeze’ and that this is more severe and less likely to be treated. Longitudinal cohort studies from the UK and Australia have reported that children with asthma, children who wheeze and children with episodes of pneumonia and/or early life respiratory infections are at significantly greater risk of chronic obstructive pulmonary disease (COPD) in later life.^25 26^ These findings suggest that schoolchildren living in informal settlements are at increased risk of impaired lung function and COPD as adults.

Of the numerous differences in the socio-economic and exposure characteristics between the Mukuru and Buruburu schoolchildren, we were able to identify adverse associations between respiratory outcomes and reported exposure to airborne pollution in general and to specific pollution sources. Although objective PM_2.5_ personal exposure based on measurements in homes, school and outdoor areas was associated with respiratory outcomes in unadjusted analyses, these were non-significant after adjustment. Possible explanations include that a single timepoint PM_2.5_ measurement is an inadequate metric of lifetime exposure, or that the source/composition of PM_2.5_ is important, for example, mosquito coils, traffic, solid fuels, burning refuse. Another possibility is that sources of particulate pollution also release aetiologically important pollutants, for example, volatile organic compounds, persistent organic pollutants and irritant gases such as nitrogen oxides and sulfur dioxide.^27–29^ It is also possible that exposure to air pollution is not the driver of the excess in asthma-related symptoms seen in the Mukuru children and that the association between symptoms and self-reported exposures sources is due to pollution sources exacerbating symptoms rather than playing any role in asthma aetiology. A study of incident asthma in South African informal settlements similarly did not find an association with measured PM_2.5_, however there was an association with NO_2_.^29^ The burning of mosquito coils has been associated with childhood asthma in LMICs.^30 31^

The lack of difference in lung function and exercise induced bronchoconstriction measured by spirometry in Mukuru and Buruburu schoolchildren is notable, particularly in the context of clear and clinically significant differences in symptoms. Methodological considerations are unlikely to have contributed to this because spirometry was conducted to international standards and children with ‘ever asthma’ had reduced FEV_1_/FVC ratio and increased EIB, suggesting our lung function measurements were valid. While the absence of any difference in spirometry between Mukuru and Buruburu schoolchildren may merely be a consequence of most children with asthma having normal lung function,^16 32 33^ it needs to be acknowledged that in an LMIC informal settlement the pathogenesis of the respiratory symptoms in children needs further investigation, including deeper phenotyping of those affected and more rigorous longitudinal exploration of lung function, including tests of small airway function.

Strengths of the Tupumue study include the number of schoolchildren recruited and the use of widely accepted methodologies and tools to investigate the prevalence of asthma symptoms in children, and that spirometry was conducted to international standards.^16 18 20 21^ An additional strength was the use of a well-described, simple non-invasive bronchial provocation study in the form of the 6 min run test to identify children with exercise-induced bronchospasm, a pathophysiological feature commonly found in children with asthma attributed to bronchial hyper-responsiveness.^19^ Particular efforts (including the use of creative arts) were taken to ensure strong involvement of both communities, in the design of communication, recruitment and data collection strategies. Fieldworkers reported that children were very aware of the study, its aims and the methods being used. Our estimates of PM_2.5_ exposure were based on AQM measurements made in the homes of 179 children, in schools, in local meeting places and at roadsides, however the modelling of total personal exposure and extrapolation to the entire sample of children is likely to have resulted in some error. Given the local concerns with air pollution, there was a possibility of response/social desirability bias in the reporting of respiratory symptoms, however this appeared not to be the case because cough symptoms did not differ between the two communities.^34^ The majority (95%) of the children participating in this study were aged 6–18 years making it highly likely that the wheezing symptoms reported were a consequence of asthma rather than viral-induced wheeze that predominantly occurs in children aged ≤5 years.^16^

The main limitation of the study is that we can only report associations because of the cross-sectional study design. A further limitation is that the widely used metric of ‘doctor diagnosed asthma’^20 21^ is probably not reliable in an LMIC context where asthma is underdiagnosed and undertreated^8^ and parents are not aware of, or are reluctant to acknowledge that their children have asthma because of widespread misconceptions and stigma.^35^ Throughout the study, it was evident that both communities were aware of, and understood the terms asthma and wheeze, possibly reflecting the high English proficiency rate in Kenya and that English is an official language, however it remains a possibility that linguistic issues may have led to under-reporting of wheeze in Mukuru schoolchildren.^36^ At the request of the communities, because of community concerns over air pollution, we conducted exploratory analyses of associations with air pollution-related exposures. However, given the diversity and complexity of the exposures encountered in informal settlements, further work will be required to investigate the potential effects of air pollution, infection, nutrition, occupation, overcrowding, housing conditions and early life. Another potential limitation is that only a minority of children attending schools in Buruburu lived locally. Most, however, lived in adjacent areas and were from more affluent homes than the children attending schools in Mukuru. Sensitivity analyses suggest that the observed differences in respiratory symptoms between Mukuru and Buruburu are underestimates.

## Conclusions

In a cross-sectional comparison, children attending schools in an informal settlement had a greater burden of symptoms consistent with asthma than children attending schools in a more affluent adjacent residential area. They were also more likely to have been exposed to higher concentrations of fine particulate air pollution. We found adverse associations between ‘current wheezing’, a symptom consistent with asthma and reported exposure to dusts, vapours, gases, fumes, adult smokers in the home, the burning of mosquito coils and refuse burning within sight of the home. These findings are pertinent to the 350–500 million children living in informal settlements worldwide.

## Data Availability

Data are available on reasonable request.
