# Supplementary material for: Asthma symptoms, spirometry and air pollution exposure in schoolchildren in an informal settlement and an affluent area of Nairobi, Kenya
Source: Thorax. 2023 Jun 6;78(11):1118–25. doi: 10.1136/thorax-2023-220057 (PMC10715514; doi:10.1136/thorax-2023-220057)
Supplement: Supplementary data [file thorax-2023-220057supp001.pdf]

BMJ Publishing Group Limited (BMJ) disclaims all liability and responsibility arising from any reliance placed on this supplemental material which has been supplied by the author(s)

Supplemental Material

Asthma symptoms, spirometry and air pollution exposure in schoolchildren in an informal settlement and an affluent area of Nairobi, Kenya

Meme H, Amukoye E, Bowyer C, Chakaya J, Das D, Dobson R, et al

Meme H, *et al. Thorax* 2023; 78:1118–1125. doi: 10.1136/thorax-2023-220057

Questions reported in the manuscript.

1. In the home where this child lives is there

2.	electricity,	(Y, N)
3.	a television,	(Y, N)
4.	a cell phone,	(Y, N)
5.	a refrigerator,	(Y, N)
6.	an indoor bath or shower,	(Y, N)
7.	an indoor tap,	(Y, N)
8.	a flush toilet,	(Y, N)
9.	a washing machine,	(Y, N)
10.	a landline telephone.	(Y, N)
11.	Does the family own a car	(Y, N)

12. Has this child had wheezing or whistling in the chest in the past 12 months? (Y / N /DK)
16. How many attacks of wheezing has this child had in the past 12 months? (None, 1-3, 4-12 >12)
17. In the past 12 months, how often, on average, has this child's sleep been disturbed due to wheezing? (Never, <1 night/week, ≥1 night/week)
18. In the past 12 months, has wheezing ever been severe enough to limit this child's speech to only 1 or 2 words at a time between breaths? (Y / N /DK)
19. Has this child ever had asthma? (Y / N /DK)
20. Was this child's asthma confirmed by a doctor? (Y / N /DK)
22. Has this child used any inhaled medicines e.g. puffers to help his/her breathing problems at any time in the past 12 months? (Y / N /DK)
24. In the past 12 months, how many times have you urgently taken this child to a doctor because of his/her breathing problems? (None, 1-3, 4-12 >12)
27. In the past 12 months, how many days (or part days) of school has this child missed because of his/her breathing problems? (None, 1-3, 4-12 >12)
29. In the past 12 months, has this child had a dry cough at night, apart from a cough associated with a cold or chest infection? (Y / N /DK)
30. Does this child ever have trouble with his/her breathing? (never, only rarely, repeatedly, but it always gets completely better, continuously, so that breathing is never quite right)

34. How many chest infections did this child have in his/her first year of life? (None, 1, 2-5, ≥6)
39. Has this child *ever* been diagnosed with pneumonia or bronchopneumonia? (Y / N /DK)

Meme H, *et al. Thorax* 2023; 78:1118–1125. doi: 10.1136/thorax-2023-220057

41. Has this child *ever* been diagnosed with TB (tuberculosis)? (Y / N /DK)
43. Has this child *ever* been diagnosed with HIV? (Y / N /DK)
44. How close is your child's home to a major road? (a road that has lorries and/or regular buses/minibuses) (<100m, 100-500m, >500 m)
48. Does anyone smoke cigarettes or tobacco inside the building where your child sleeps? (Y / N /DK)
49. Does your child smoke cigarettes or anything else? (Y / N /DK)
50. In your child's everyday life, does he/she breathe in vapours, dusts, gases or fumes for more than 15 hours per week? (Y / N /DK)
51. Is there ever a fire to burn refuse (waste, rubbish) within sight of your home? (Y / N /DK)
52. Do you burn mosquito coils at home? (Y / N /DK
53. This section is about the main stoves/devices that are used at your home (including cooking food, making tea, and boiling drinking water), thinking about the one that is used most often in your home What type of cookstove is it? (Electric cooker, Solar cooker, Gas cooker, Liquid fuel cooker, Solid fuel cooker, Three stone stove / open fire, Other)

Meme H, *et al. Thorax* 2023; 78:1118–1125. doi: 10.1136/thorax-2023-220057
